# Investigation of Dual-Mode Microstrip Bandpass Filter Based on SIR Technique

**DOI:** 10.1371/journal.pone.0164916

**Published:** 2016-10-31

**Authors:** Yaqeen S. Mezaal, Jawad K. Ali

**Affiliations:** 1 Electronic and Communication Engineering Department, Cankaya University, Ankara, Turkey; 2 Computer Engineering Techniques Department, Al-Esraa University College, Baghdad, Iraq; 3 Microwave Research Group, Electrical Engineering Department, University of Technology, Baghdad, Iraq; West Virginia University, UNITED STATES

## Abstract

In this paper, a new bandpass filter design has been presented using simple topology of stepped impedance square loop resonator. The proposed bandpass filter has been simulated and fabricated using a substrate with an insulation constant of 10.8, thickness of 1.27mm and loss tangent of 0.0023 at center frequency of 5.8 GHz. The simulation results have been evaluated using Sonnet simulator that is extensively adopted in microwave analysis and implementation. The output frequency results demonstrated that the proposed filter has high-quality frequency responses in addition to isolated second harmonic frequency. Besides, this filter has very small surface area and perceptible narrow band response features that represent the conditions of recent wireless communication systems. Various filter specifications have been compared with different magnitudes of perturbation element dimension. Furthermore, phase scattering response and current intensity distribution of the proposed filter have been discussed. The simulated and experimental results are well-matched. Lastly, the features of the proposed filter have been compared with other designed microstrip filters in the literature.

## Introduction

Filtering Techniques have been employed in all fields of signal processing to handle the features of electrical signals [1–2] or organize signal transmission among specific frequency bands [3].

Microstrip bandpass filters are a particular type of filters that can be used to pass a definite range of frequencies and decline another one based on their application requirements. These filters are typically realized using one or more resonators, coupled to each other. These resonator components represent physical elements that store both electric and magnetic energy in a frequency-dependent way. As compared to waveguide filters, microstrip filters have smaller sizes, but in several applications very compact microstrip filters are required. Currently, radar, mobile and satellite communication systems are application examples which necessitate miniature filters as imperative components [4]. Certainly, besides the compactness requirement, other parameters in the filter design must be taken in the consideration. For instance, high return loss, small insertion loss and big rejection band levels. These represent the features of a high-quality filter. Although the microstrip filter miniaturization can be realized from dielectric substrates with big magnitudes of dielectric constant, size reduction by the change of filter geometry has more preference since huge dielectric constant materials will frequently launch additional losses and surface waves. Some practical methods can be used to carry out a reduced size filter design such as the process of bending different parts of the filter. This possibly will be the most advantageous process to obtain more compact sizes, especially for extensive straight transmission lines or stubbed filters. Ultra-wideband filters stated in [5] and wideband filter stated in [6] are the filter instances that use line bending method.

Alternatively, miniaturized microstrip filters can be constructed by using dual-mode resonators. The most important benefit of these categories of the resonator is that each dual-mode resonator operates as a dual tuned resonant circuit and then, an *n*-degree filter can be accomplished in smaller arrangements and less complicated owing to the halved resonator numbers [7]. Theoretically, dual mode filters can be classified to degenerate and non-degenerate devices. In most cases, a small patch or slit as perturbation element is inserted into the dual degenerate mode resonator at 45° offset from its orthogonal input/output feeders to generate a frequency response with degenerate transmission poles [7]. On the other hand, non-degenerate dual-mode filters typically use non-orthogonal input/output feeders without inserting perturbation element as compared to dual degenerate mode filters. Also, these non-degenerate filters have been designed with larger fractional bandwidth up to 25% than dual degenerate ones with a typical fractional bandwidth less than 5% [8]. Nevertheless, many research interests about designing more compact dual-mode filters with diverse electrical measurements are still in progress [7, 8].

Dual-mode microstrip bandpass filters based on 2^nd^ and 4^th^ order configurations have been presented in [9]. These filters are designed using square-loop resonator and non-orthogonal input/output feeders positioned along a straight line on the frequency response. All filters exhibit two transmission zeros in their output frequency responses. Also, a full analysis about the effect of non-orthogonal input/output feeders and resultant transmission zeros have been achieved using Sonnet simulator.

In [10], two bandstop filters using dual-mode square loop resonators and bent λ/4 microstrip feeding line have been simulated, fabricated and measured. Both filters have degenerated frequency responses using a capacitive or inductive perturbation component at one of its corners. The coupling coefficients and perturbation effect have been analyzed for these filters.

A new dual-mode microstrip bandpass filter to generate dual-band frequency response has been reported in [11]. This filter uses resonator structures based on a couple of open-circuited stubs to the corner points of a square loop resonator. Through the movement of the gaps between the open ends to obtain a perturbation effect, dual degenerate modes are induced, while transmission zeros and mode frequencies are controllable to acquire the most suitable frequency response. Simulation and measurement frequency responses are in good agreement. In [12], novel microstrip bandpass filters using dual-mode square loop resonator with mitered bends have been designed. A parametric study to determine the effect of narrow slits on filter responses has been conducted in this study. All filters have elliptical and linear phase frequency characteristics. The simulated and measured results are well-matched to each other. Two dual-mode microstrip bandstop filters have been designed using square loop resonators as 4^th^ order configuration as stated in [13]. The position of reflection zeros and degenerated modes can be straightforwardly adjusted by changing the perturbation element size. Both filters have dual reflection zeros in their output frequency responses that are verified with compatible simulations and measurements. Dual-mode meandered loop resonator has been employed to simulate and fabricate dual-mode dual-band microstrip bandpass filters as reported in [14]. The center frequency tuning can be controlled by varying the connection points within square loop loading elements without varying the entire surface area. The simulated frequency responses for the designed filter have elliptical and linear phase filtering characteristics that are in good agreement with the measured ones. New bandpass filter using dual-mode slotted square patch resonator has been reported in [15]. This filter uses orthogonal input/output feeders and corner perturbation element to generate a frequency response with dual transmission zeros around a center frequency of 2 GHz. Full parametric study about slotted patch filter as compared to classical square patch filter has been achieved using MWO simulator.

The proposed microstrip bandpass filter in this paper is based on dual degenerate mode resonator. This resonator has been constructed by applying SIR approach on all sides of the closed square loop resonator. The designed bandpass filter has very miniature surface area (less than 1cm^2^) that can be integrated within any wireless device or communication system. The output frequency response of this filter has quasi elliptic characteristics as well as interesting electrical specifications. The performance of this device has been demonstrated by fabrication and measured results. Also, the proposed filter in this study has been compared with other reported works in the literature.

## Step Impedance Resonator

Stepped Impedance Resonator (SIR) approach can be defined as Transverse Electromagnetic (TEM) or quasi TEM mode transmission line resonator that has two or more lines with different characteristic impedances. This method has the advantage of being relatively easy to build. Additionally, it takes less size as compared to a similar low-pass filter using stubs. The main disadvantage is the relative degradation of electrical performance. This method of construction is often indicated for applications where a sharp cutoff frequency is not required. More specifically, because of the radiations, transverse resonances and other undesirable effects, SIR method is not suitable for microwave frequencies higher than 20 GHz. However, for frequencies beyond 20 GHz, this disadvantage becomes a minor issue with recent technologies and simulators available to engineers [16].

**Fig 1:**
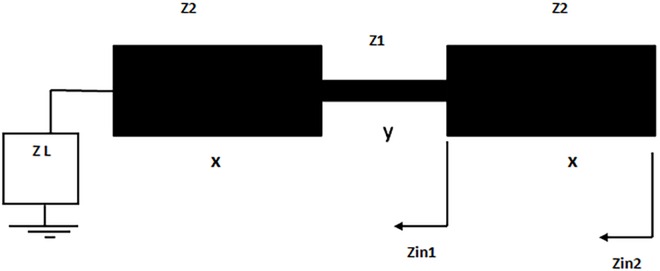
Schematic of the adopted SIR approach in this study.

The employed SIR technique in this research article is depicted in **Fig 1.** It represents the transmission line model of the symmetric SIR which is formed by low and high impedance sections (Z1 and Z2). Since it is, as depicted in **Fig 1**, the resonant modes of the designed SIR can be modeled and evaluated by the transmission line theory. The input impedance (*Zin*1) viewed at the junction of high impedance section to low impedance section can be determined by [17]:

$$Zin1=jZ1\frac{Z2\;\tan\;x+Z1\;\tan\;y}{Z1-Z2\;\tan\;x\;\tan\;y}$$ (1)

where *x* and *y* are electrical lengths of the high and low impedance sections, respectively. Moreover, the input impedance (*Zin*2) viewed at the right terminal of the SIR transmission line can be determined by [17]:

$$Zin2=Z2\frac{Zin1+jZ2\;\tan\;x}{Z2+jZin1\;\tan\;x}$$ (2)

The even and odd mode frequencies of the stepped impedance square loop resonator occur when *ZL* equals to 0 and *∞* respectively [17]. On the other hand, the fundamental frequency can be controlled by scaling the electrical lengths for x and y sections of the adopted resonator [18].

SIR is a non-standardized transmission line, which can be adopted in the filter design either for miniaturization objectives, or to reallocate the spurious pass-band to the upper frequency, or to isolate the harmonic frequencies [18]. In this study, SIR approach has been used to isolate 2^nd^ harmonic frequency in output frequency response of the filter.

## Filter Design and Results

**Fig 2:**
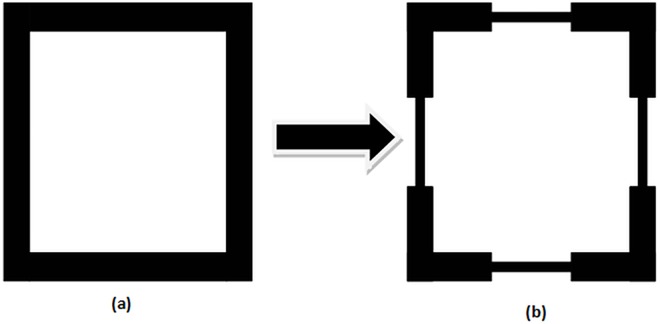
Microstrip resonator transformation; (a) The classical square loop resonator reported in [7] and (b) Stepped impedance square loop resonator that adopted in the planned filter of this study.

The planned microstrip bandpass filter layout is fundamentally derived from classical dual-mode square loop resonator reported in [7]. The bandpass filters using this classical resonator have often 2^nd^ harmonic frequency in their frequency responses that attenuates the electrical signal in their pass-band regions. The main objective here is to present a new filter design with compact size, better selectivity and second harmonic frequency suppression by applying SIR approach depicted in **Fig 1** on all sides of the classical square loop resonator. **Fig 2** explains the microstrip resonator transformation from the classical square loop resonator to stepped impedance square loop resonator for the filter construction in this study.

The designed bandpass filter has been tested and optimized using Sonnet simulator. This simulator performs electromagnetic investigation using the full wave electromagnetic principle. The simulator has been adjusted under conditions of 0.1mm grid size modeling and 0.025 GHz step frequency for different frequency ranges in this study. Also, all applied dimensions are in mm units.

**Fig 3:**
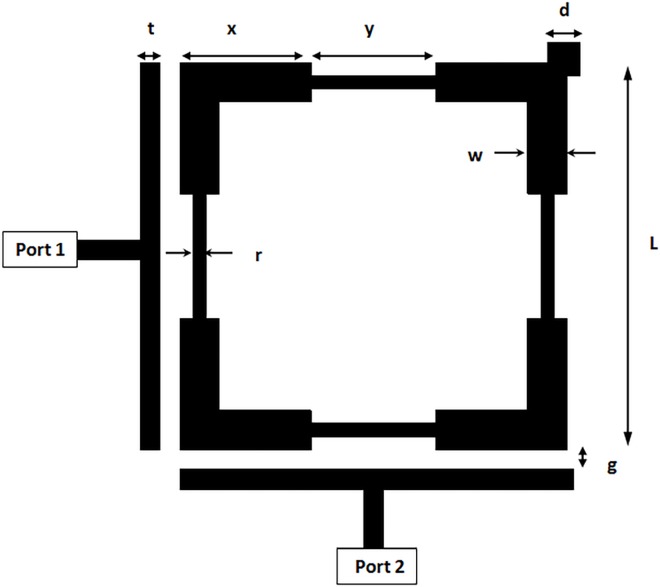
The configuration of stepped impedance square loop resonator bandpass filter.

The proposed filter uses stepped impedance square loop resonator and orthogonal input/output feeders with up right corner perturbation element as depicted in **Fig 3**. The layout of this filter has been modeled on a substrate with a relative dielectric constant (*ε*_*r*_) of 10.8 and a thickness (H) of 1.27 mm. The resonator side length (L) is 5.9 mm with w = 0.6 mm, x = 2 mm, y = 1.9 mm and r = 0.2 mm. The spacing (g) between input/output feeders and the resonator has been adjusted to 0.2 mm. The perturbation element side length (d) in the up right corner of the resonator has been set to 0.5 mm. On the other hand, the width of input/output feeders (t) has been set to 0.3 mm.

**Fig 4:**
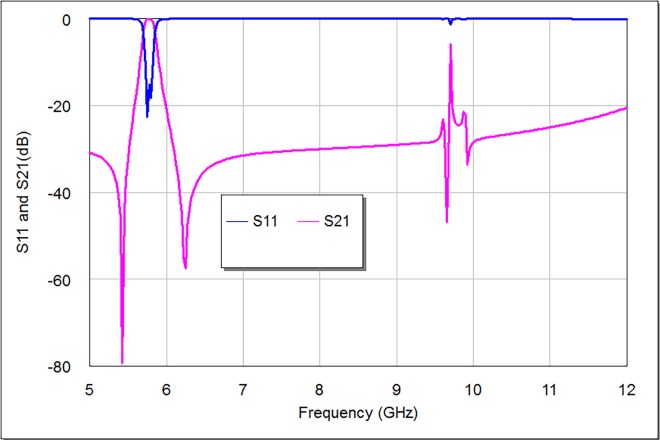
The transmission and return loss responses of the designed bandpass filter.

The simulation results of return loss (S11) and transmission (S21) responses of this filter are depicted in **Fig 4**. It is comprehensible by this graph, that the modeled bandpass filter presents a quasi-elliptic transmission response with dual transmission zeros which are asymmetrically situated around deign frequency near pass-band edges. The modeled filter has 5.8 GHz center frequency with dual transmission poles at 5.75 and 5.81 GHz respectively. The magnitudes of return loss and insertion loss are 22.578 and 0.134 dB respectively. The simulated filter bandwidth is 150 MHz within 3 dB pass-band region, which represents 2.586% of its center frequency. The transmission zeros are originated from middle top and bottom arms of stepped impedance square loop resonator of the modeled filter, while the transmission poles are originated from middle left and right arms of the same filter.

In addition to center frequency, the most important electrical requirements of any bandpass filter are insertion loss and return loss [7]. Insertion loss, expressed in decibels (dB) unit, is frequently used within telecommunication systems to resolve the terminal attenuation that generates from inserting a filter into the signal path. Theoretically, insertion loss is preferred to be less than 1 dB. On the other hand, return loss is a determination of the voltage standing wave ratio. It is also expressed in dB unit. It is attributed to the impedance mismatches between the circuits. A big magnitude of return loss (preferably higher than 10 dB) means a higher quality of the filter under test. Accordingly, the insertion loss and return loss magnitudes of the proposed filter are very suitable to be integrated within many wireless systems or communication devices.

**Fig 4** also explains the transmission and return loss responses of the designed filter after pass-band region. It is apparent that the frequency responses don’t support a second harmonic frequency (2*f)* around 11.6 GHz that characteristically appears in the classical bandpass filter responses. There is a noticeable spurious response in this graph around 9.7 GHz. However, it is not effective since it is obviously isolated as shown in **Fig 4**.

**Fig 5:**
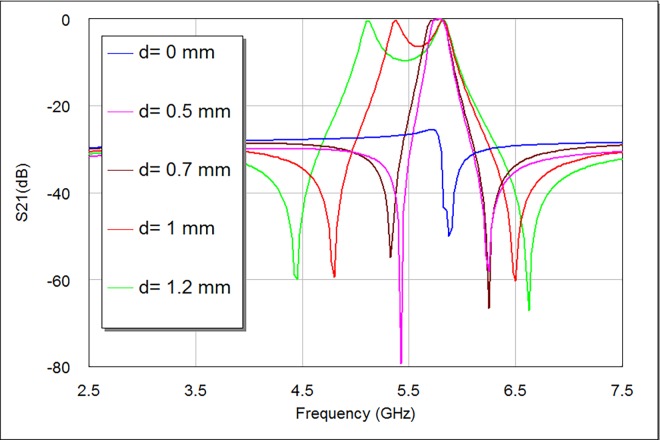
S21 responses of proposed bandpass filter as a function of d.

**Fig 6:**
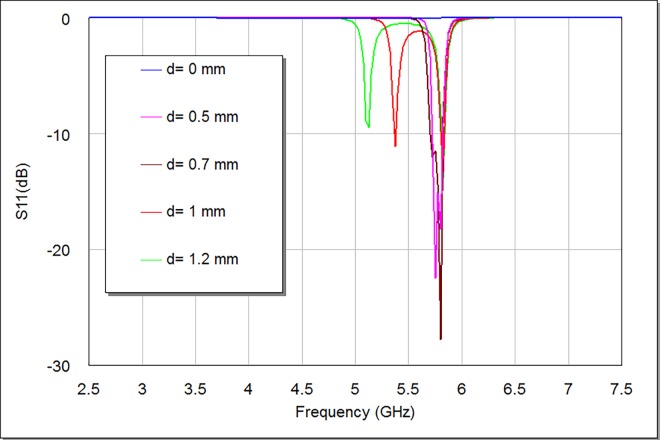
S11 responses of the proposed bandpass filter as a function of d.

A parametric investigation has been prearranged to inspect the importance of d parameter on the modeled bandpass filter responses. **Figs 5 and 6** give details about S21 and S11 filter responses as a function of d.

From **Fig 5**, the increasing magnitude of d causes S21 response firstly move towards the ideal 0 dB point and then divide into dual identifiable peaks. At d = 0 mm, there would be poor S21 and S11 responses since there is no coupling between the two modes by a perturbation element. **Table 1** depicts the simulated electrical specifications of the designed filter with d magnitudes (0, 0.5, 0.7, 1, and 1.2) mm. It is perceptible, from **Figs 5 and 6** and **Table 1**; that the discrepancy in d magnitude somewhat influences the resonant frequency, whereas its effect is clearer on the return loss, transmission zeros, insertion loss, bandwidth and mode resonances. The minimum perturbation length to produce a reasonable frequency response has been found at d = 0.5 mm, which is as well the most suitable response to be adopted here at design frequency of 5.8 GHz. Also, this d parameter is noticeably proportional to bandwidth and insertion loss magnitudes, while it is to some extent inversely proportional to return loss magnitudes as they are recognizable by **Table 1**. There are similar odd resonances at d = 0.5 and 0.7 mm as well as at d = 1 and 1.2 mm, which give the impression that d parameter mostly affect even resonances as compared to odd ones. It is noticeable from S21 responses in **Fig 5** that d magnitude affects the position of left transmission zeros slightly more than the right ones.

**Table 1:** The electrical parameters of the proposed filter with respect to d magnitudes.

Parameters	d = 0 mm	d = 0.5 mm	d = 0.7 mm	d = 1 mm	d = 12 mm
Best S11 in Passband Region (dB)	……….	22.578	27.79	11.07	9.5
Insertion Loss (dB)	……….	0.134	0.24	6.364	9.618
Center Frequency(GHZ)	……….	5.8	5.83	5.6	5.474
Bandwidth(MHz)	………	150	154	546	805
Left and Right Transmission Zeros(dB)	………	79.35, 57.98	54.86, 66.57	59.48, 60.32	59.92, 67.11
Even and Odd Mode Resonances(GHz)	………	5.7,5.85	5.66, 5.85	5.326, 5.872	5.067,5.872

In addition to d parameter, the edge spacing (g) between the resonator and input/output feeder, can be used as secondary tuning limitation to increase return loss and decrease insertion loss of the filter response as well as possible. Also, choosing input/ output port positions can enhance the isolation of 2^nd^ harmonic frequency for the proposed filter.

An imperative subject in the compactness of microstrip filters is that all resonating devices must involve certain dimensions in terms of λ_*g*0_, which can be calculated at the operating frequency (*f*) by [7]:

$$\lambda_{g0}=\frac{c}{f\sqrt{\varepsilon_{e}}}$$ (3)

$$\varepsilon_{e}\approx \frac{\varepsilon_{r}+1}{2}$$ (4)

where c and *ε*_*e*_ represent light speed and effective dielectric constant respectively. There are possibly better approximations for *ε*_*e*_, however the additional efforts to obtain more exact *ε*_*e*_ are still not worth it [19]. From these equations, *ε*_*e*_ = 5.9 and λ_*g*0_ = 22.294 mm have been calculated at *f* = 5.8 GHz. So, by these computations, the dimensions of the proposed dual mode resonator without input/output feeders in term of λ_*g*0_ have been found to be 0.277 λ_*g*0_ x 0.277λ_*g*0_, which are theoretically tolerable for microwave filters. The term of λ_*g*0_ can be used to compare the areas of various resonating devices under different magnitudes of resonant frequency, dielectric constant, dielectric thickness and resonator dimensions.

**Fig 7:**
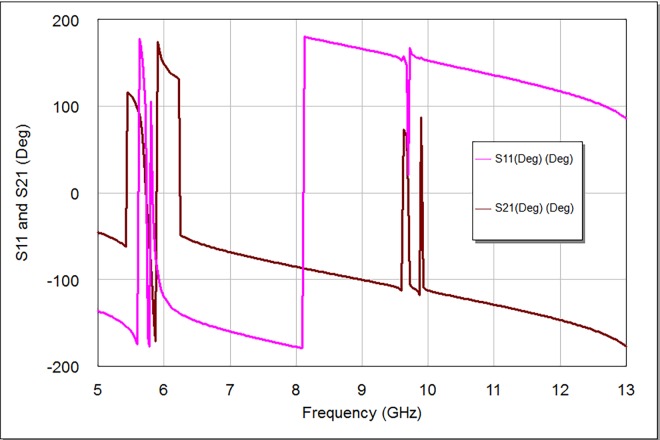
The phase response of the proposed bandpass filter.

**Fig 7** shows the S21 and S11 phase scattering curves within frequency range from 5 to 13 GHz and within output phase angle from -200 to 200 degrees. These resultant curves offer several bouncy phases ranged from -175 to 180 degrees for S11 and S21 scattering curves. Consequently, the intersections between S21 and S11 responses are noticeable; especially around design frequency of 5.8 GHz and spurious response around 9.7 GHz.

**Fig 8:**
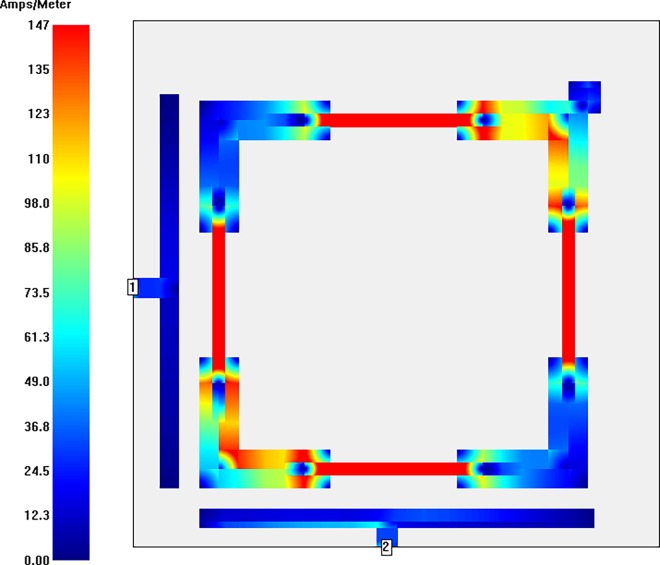
Current intensity distribution of stepped impedance square loop resonator bandpass filter with perturbation element at 5.8 GHz.

**Fig 9:**
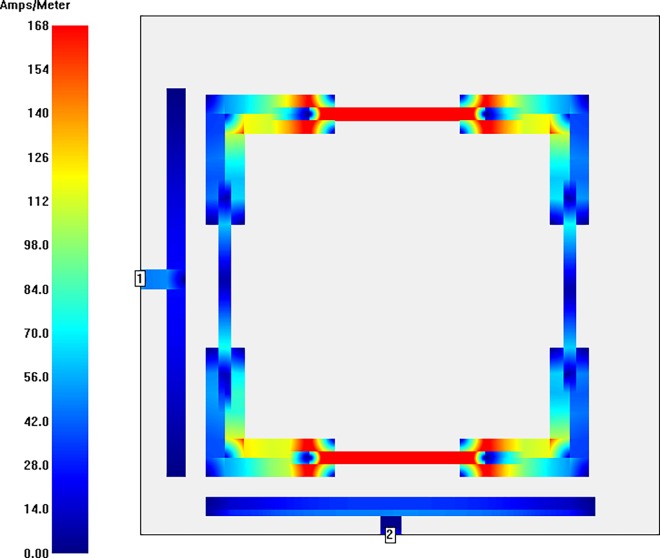
Current intensity distribution of stepped impedance square loop resonator bandpass filter without perturbation element at 5.8 GHz.

With the aim of getting closer to the nature of current distributions of the modeled bandpass filter, the simulated surface current intensity distributions at a center frequency of 5.8 GHz with and without perturbation element are illustrated in **Figs 8 and 9** using Sonnet simulator. The highest coupling outcome is represented by red color while the smallest one is observable by blue color. It is obvious from these current distributions at the center frequency that the current intensity in the microstrip filter is symmetrically patterned. The strongest current distribution can be observed at all y sections with magnetic intensity of 147 Ampere/Meter for filter structure with perturbation element. On the other hand, the maximum intensity is 168 Ampere/Meter in the up and bottom y sections of the filter structure without perturbation element. This, of course, denotes to the second harmonic frequency isolation due to the applied SIR technique. Also, it is recognizable that the filter structure including perturbation element has less magnetic intensity than the filter structure without perturbation element.

**Fig 10:**
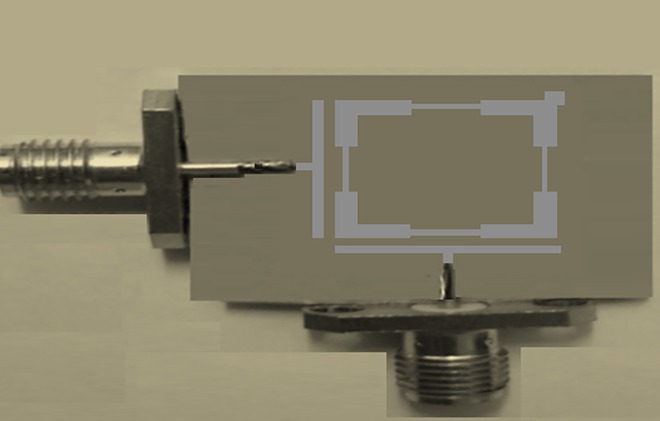
The fabricated bandpass filter snapshot.

The proposed filter has been manufactured using RT/Duroid substrate of 10.8 insulation constant, 1.27 mm substrate thickness, 0.0023 loss tangent and conductor thickness of 35 μm. **Fig 10** illustrates the snapshot of the fabricated bandpass filter. From this figure, dual 50Ω SMA ports have been connected to 50Ω feeders to join to the Vector Network Analyzer (VNA) for better experimental measurements.

**Fig 11:**
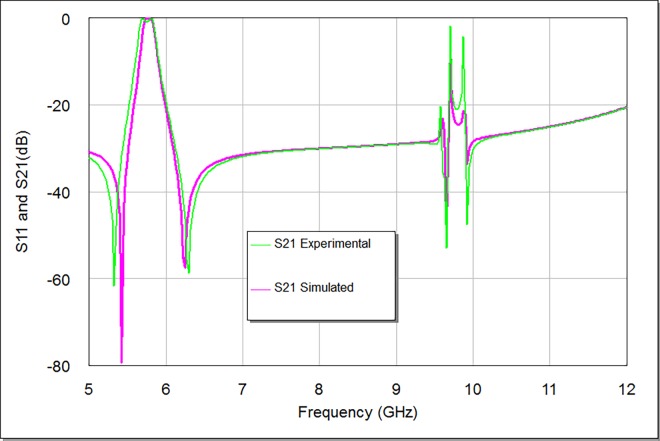
Simulated and experimental S21 responses.

**Fig 12:**
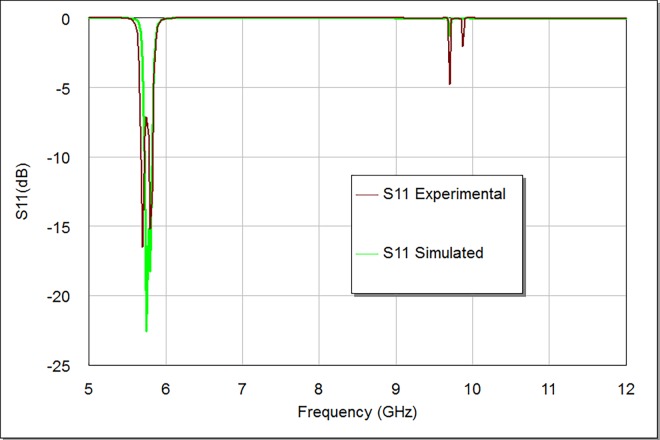
Simulated and experimental S11 responses.

**Figs 11 and 12** illustrate the simulation and experimental frequency results of the designed filter. The measured return loss is 16.63 dB and the smallest amount of insertion loss of the filter is better than 1 dB, while the measured bandwidth is 200 MHz. The comparative filter specifications of simulation and measurement are shown in **Table 2**. There are acceptable deviations between simulated and measured results; especially for return loss, insertion loss and bandwidth magnitudes. These deviations are mainly attributed to conductor loss, connector mismatches and fabrication tolerance. The coupling gap values between input/ output feeders and stepped impedance square loop resonator in addition to etching process are important factors that can be used to decrease the insertion loss to its minimum. Also, in the adopted design, we used a suitable substrate material with good dielectric constant and conductor thickness to enhance the filter performance in terms of compactness and insertion loss in the pass-band region. It is feasible to boost the filter response by using good input/ output feeder configurations with appropriate feeder coupling space, length and width.

**Table 2:** Comparison of the simulation and experimental results for the proposed filter.

Filter Parameters	Simulated	Measured
Center Frequency (GHz)	5.8	5.8
Insertion Loss (dB) at Center Frequency, S21	0.134	0.95
Best Return Loss (dB) in Passband, S11	22.578	16.63
Bandwidth (MHz)	150	200

In addition to design simplicity, the measured results of this filter have almost better performance in terms of insertion loss, return loss and bandwidth than microstrip filters reported in [20, 21] under similar center frequency as it can be observed from **Table 3**. Also, the presented filter design of this study has a miniature surface area of 0.81cm^2^, which is appropriate to be integrated within many wireless systems or communication devices. Regardless of design frequency and electrical filter specifications, the surface area of this filter is smaller than all designed filters reported in [4–6, 8–21].

**Table 3:** Comparison of the experimental results for the proposed filter with reported filters in [20] and [21] at 5.8 GHz center frequency.

Filter Parameters	Our Work	Bandpass filter reported in [20]	The narrow band bandpass filter state reported in [21]
Insertion Loss (dB), S21	0.95	3.2361	1.821
Best Return Loss (dB) in Passban, S11	16.63	14.502	18
Bandwidth(MHz)	200	750	255
H,*ε*_*r*_ (Substrate Specifications)	1.27 mm, 10.8	1.6 mm, 4.4	50 mils, 6.15
Design Complexity	The simplest	More Complicated	The Most Complicated

## Conclusion

New dual degenerate mode microstrip bandpass filter based on SIR approach on all sides of the square loop resonator has been presented in this paper. The designed filter has been constructed from a dielectric material with an insulation constant of 10.8 and a thickness of 1.27mm to be applied at 5.8 GHz design frequency. This filter has very small surface area as well as sufficient transmission and return loss magnitudes with an isolated second harmonic frequency that represent the most requested features to be applied in any wireless application. Both simulated and measured results of the designed filter are in good compatibility to each other. Parametric study about the effect of perturbation element dimension (d) on the filter response has been investigated. Additionally, phase scattering response and current intensity distribution for the proposed filter have been considered. At the same design frequency, the proposed filter in this study has almost better electrical specifications than reported works in [20] and [21]. Also, it has smaller surface area than all reported filters in [4–6, 8–21], regardless of design frequency and electrical filter measurements.
